# Novel Scent Enrichment Enhances Socio‐Sexual and Olfactory Behaviors in Zoo‐Housed Gentle Lemurs

**DOI:** 10.1002/ajp.23716

**Published:** 2024-12-18

**Authors:** Sara Fontani, Gale Glendewar, Rachel Cowen, Georgia Callagan, Anna B. Costantini, Emily Elwell, Colin Dubreuil, Matthew Palframan, Stefano Vaglio

**Affiliations:** ^1^ School of Life Sciences, Animal Behaviour & Wildlife Conservation Group University of Wolverhampton Wolverhampton UK; ^2^ Durrell Wildlife Conservation Trust – Jersey Zoo Trinity Jersey Channel Islands; ^3^ Department of Biosciences University of Milan Milan Italy; ^4^ School of Pharmacy University of Wolverhampton Wolverhampton UK; ^5^ University College–The Castle, Durham University Durham UK

**Keywords:** captive breeding, captive welfare, chemical signalling, cortisol, fecal endocrinology, *Hapalemur alaotrensis*, scent enrichment, testosterone

## Abstract

The Alaotran gentle lemur (*Hapalemur Alaotrensis*) is one of the most endangered primates in the world and shows a low success rate in captive breeding programmes. We tested a novel scent enrichment, made up of a synthesized mixture likely conveying information about female fertility, on four unsuccessful breeding pairs (*n* = 8 subjects) living at the Jersey, Birmingham, London (United Kingdom) and Mulhouse (France) zoos. We evaluated the effects of the scent enrichment on behavior (515 h of observation) and fecal endocrinology (cortisol and testosterone measurements) (*n* = 180 samples) comparing pre‐ enrichment, enrichment and post‐ enrichment phases. We found a small effect by sex on olfactory behaviors related to the enrichment. We also found that both male and female sexual behaviors significantly increased during the enrichment. Conversely, we did not find any significant change related to enrichment in cortisol and testosterone levels, whilst some effect by the zoo environment. Our results show little effectiveness by the scent enrichment as the lemur hormone levels did not change significantly and the lemurs continued to fail to reproduce following the enrichment. Nevertheless, our findings highlight that biologically relevant scent may trigger natural species‐specific behaviors, with potential implications for enhancing behavioral health and management of zoo‐based endangered lemur species.

## Summary

- Novel scent enrichment triggered olfactory and sexual behaviors in zoo‐housed, critically endangered lemur species.
- Despite limited impact on hormone levels, the scent enrichment revealed potential for eliciting species‐specific behaviors which could be crucial for managing captive breeding programmes of endangered zoo species.
- Findings suggest that biologically‐relevant scents could play a pivotal role in enhancing natural species‐specific behaviors, with implications for the conservation and management of captive populations of endangered species.

## Graphical abstract

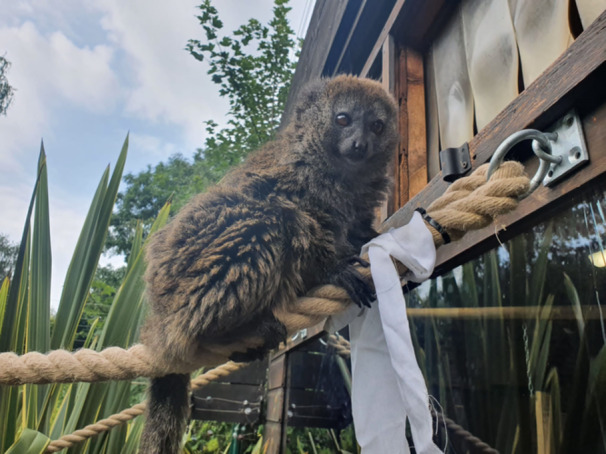

## 1 Introduction

With almost 60% of primate species currently classified as endangered or critically endangered by the International Union for Conservation of Nature (IUCN) (IUCN 2022), primate conservation is now of vital importance (Estrada et al. 2017). Several European Association of Zoos and Aquaria (EAZA) institutions are involved in conservation initiatives that integrate in situ and *ex situ* programmes, which aim to improve the conservation status of both the target species and other species living in the same environment (Lacy 2009; Spiezio et al. 2022). The maintenance of the genetic variation of zoo populations, especially those involved in captive breeding (e.g., EAZA Ex‐situ Programmes (EEP)) and reintroduction programmes, plays a pivotal role in fighting biodiversity loss (Britt et al. 2004; Lacy 2009; Schulte‐Hostedde and Mastromonaco 2015). However, several endangered primate species currently shows a low success rate in captive breeding and so cannot provide offspring for reintroduction into the wild, which impair them from possibly serving as a buffer against extinction (reviewed in Elwell and Vaglio 2023). For example, captive populations of Alaotran gentle lemur (*Hapalemur alaotrensis*) would not be able to support reintroduction actions into the wild due to lack of individuals (Spiezio et al. 2022).

The need of captive animals to express natural behaviors has been acknowledged by many studies (e.g., McPhee and Carlstead 2010; Wielebnowski 1998). In the zoo environment, the lack of stimuli and the repetitive routine can lead to boredom (McPhee 2002), stereotypic behavior (Swaisgood et al. 2003), and endocrinological dysfunction (Jacobs et al. 2021), which may be linked with decreased reproductive fitness for captive populations (Carlstead and Shepherdson 1994; Fritz et al. 1992; Mallapur 2008; Vaz et al. 2017). Nevertheless, captivity is a human‐controlled environment and, therefore, captive breeding success may be enhanced by stimulating reproductive behavior through environmental enrichment (Ben‐Ari 2001; Carlstead and Shepherdson 1994; Moreira et al. 2007). Environmental enrichment can be described as motor, cognitive, sensory, and social stimulation that boosts animals' psychological and physiological welfare by promoting a wide range of natural species‐specific behaviors (Ben‐Ari 2001; Cummins et al. 1977), which may ultimately improve breeding success (Carlstead and Shepherdson 1994).

Primates are generally considered microsmatic mammals, relying more on visual and vocal rather than olfactory cues (Heymann 2006). However, olfactory signalling plays a crucial role in socio‐sexual communication for strepsirrhine primates (Colquhoun 2011; Drea 2015). For instance, scent‐marking behavior (i.e., the release of semiochemical signals on a substrate (Andersen 1999)) may have numerous functions (Vaglio et al. 2016), where the secreted signal can convey information about age, rank, reproductive status, diet, individual and group identity (Brahmachary and Poddar‐Sarkar 2015; Soso and Koziel 2017). In many mammal species, sexual pheromones may advertise female fertility and elicit male behavioral and physiological responses (Coombes, Stockley, and Hurst 2018). These reactions include primate species showing courtship displays aimed to facilitate attraction and mating, such as rhesus monkeys (*Macaca mulatta*) and even humans (*Homo sapiens*: Grammer, Fink, and Neave 2005). Odour may also provide females with information about quality of males as potential mates (e.g., strepsirrhine primates: Campbell‐Palmer and Rosell 2011; cheetahs, *Acinonyx jubatus*: Tommasi et al. 2023). Some chemicals, therefore, have great potential as tools for triggering olfactory and sexual behaviors in lemur species (Campbell‐Palmer and Rosell 2011). Although evidence shows that scents can facilitate mate choice and improve the chances for reproductive success in some mammals (e.g., striped dunnarts, *Sminthopsis macroura*: Parrott, Nation, and Selwood 2019; harvest mice, *Micromys minutes*: Roberts and Gosling 2004), studies on the effects of olfactory enrichments in primates are still scarce (Elwell and Vaglio 2023). Furthermore, most studies have focused on anthropogenic scents, such as spices or essential oils, rather than testing biologically relevant scents (Wells et al. 2007), which in turn may also positively impact reproductive success (Rafacz and Santymire 2014).

Our study focused on the Alaotran gentle lemur, which has an estimated population of around 2,500 individuals in the wild (Reibelt et al. 2019) and is currently listed on the Appendix I of the Convention on International Trade in Endangered Species of Wild Fauna and Flora (CITES) as among the most endangered species within CITES‐listed animals and plants. Alaotran gentle lemurs live in small troops that do not show seasonal variation in size or composition (Mutschler 2002). These units primarily consist of a mated pair and their offspring. Nonetheless, larger groups composed of multiple adults are not uncommon, varying in size from two to nine individuals (Mutschler 2002). Territory‐wise, these lemurs assert dominance over areas spanning approximately 2.5–5 acres (Guillera‐Arroita et al. 2010). Male lemurs take charge of safeguarding their territory against rival groups, yet these confrontations seldom escalate to aggressive physical encounters (Nievergelt, Mutschler, and Feistner 1998). Similar to numerous other lemur species, females are dominant, with over 90% of conflicts centred around food resources (Waeber and Hemelrijk 2003).

The reproductive patterns of Alaotran gentle lemurs predominantly lean towards monogamous relationships, although instances of polygyny do occur. Most mating takes place within the group, although around 8.5% of offspring are sired by extra‐group males (Nievergelt et al. 2002). Alaotran gentle lemurs are seasonally polyestrous, with mating occurring over 1 day per sexual cycle (Haring and Davis 1998). Mating occurs during the dry season in the wild, leading to births during the wet season, typically between September and February (Mutschler, Nievergelt, and Feistner 2000). Nevertheless, in captivity these lemurs do not show a clear breeding season (Beattie and Feistner 1998; ZIMS 2024) and beyond mating, there is no discernible variation in behavioral patterns that could be regarded as indicative of female fertility (Fontani et al. 2022; Haring and Davis 1998). Females, enduring a gestation period of roughly 140 days, often produce single offspring annually, although twins are common (Mittermeier et al. 2010).

Alaotran gentle lemurs rely on both scent and vocalizations for communication, wherein olfactory signalling assumes a dominant role in their socio‐sexual interactions. Equipped with a functional vomeronasal organ, these lemurs explore scents using both olfactory and gustatory means, displaying a remarkable sensitivity to chemically encoded messages (Drea 2020; Fontani et al. 2022; Janda et al. 2019; Scordato and Drea 2007; Wyatt 2003). While olfactory communication in this species has not been studied extensively, our recent work indicates that anogenital odour may play an important role in encoding information about fertility in female Alaotran gentle lemurs (Fontani et al. 2022).

The decline of the Alaotran gentle lemur's breeding population is likely due to management decisions made in the 1990s and 2000s as well as captive breeding failures. Since 1990 the captive population of this lemur species has been managed by an EEP (Beattie and Feistner 1998) to enhance the survival chances of this critically endangered species. However, the current EAZA gentle lemur population consists of only around 70 adult individuals and breeding has recently declined in many European zoos to only a few active breeding pairs (ZIMS 2024). In this context, improving our understanding of the Alaotran gentle lemur's reproductive biology and enhancing their well‐being and breeding success in captivity is crucial to the species' survival (Fontani et al. 2022).

The overarching aim of our study was to design and test a new scent enrichment to enhance the well‐being and reproductive potential of captive Alaotran gentle lemurs. To achieve this aim, we investigated the chemical profile of the anogenital odour secretions of a successfully breeding female (Fontani et al. 2022), then reproduced the chemical mixture in our semiochemistry laboratory. Next, we presented the new chemical mixture to four unsuccessful breeding pairs (*N* = 8 subjects) to stimulate sexual behavior. Specifically, we tested three conditions—pre‐enrichment (i.e., before enrichment); enrichment (i.e., during enrichment); posenrichment (after enrichment)—by recording behavior (focusing on aggressive, abnormal and self‐directed, olfactory, and sexual behaviors) and collecting fecal samples (to assay for stress and sex hormones).

In this study, we tested the following hypotheses and predictions:

1. Our newly designed scent enrichment would reduce stress levels. Specifically, we predicted that stress‐related behaviors (i.e., abnormal, self‐directed and aggressive) would decrease (Damasceno et al. 2017; Spiezio et al. 2021), species‐specific behaviors (i.e., olfactory) would increase (Gronqvist et al. 2013; Wells and Egli 2004), and stress hormone levels (i.e., cortisol concentrations) would decrease after the enrichment phase (Ahsan et al. 2024).
2. Our newly designed scent enrichment would trigger sexual behavior and hormones. We expected that males and females would react differently to the enrichment. Specifically, we predicted that male sexual behaviors (including mating behaviors) and male sex hormone levels (i.e., male testosterone concentrations) would increase during and after the enrichment phase (Elwell, Fontani, and Vaglio 2024; Ziegler et al. 2005), while females would display less of a response, as our enrichment is based on the female fertility signal.

## 2 Materials and Methods

### 2.1 Subjects Housing

We studied four unsuccessful captive breeding pairs (i.e., biologically able to reproduce but never successful bred as a pair due to lack of mating behavior) of Alaotran gentle lemurs (*N* = 8 subjects), hosted at Birmingham Wildlife Conservation Park (UK), Parc Zoologique & Botanique de Mulhouse (France), Jersey Zoo (formerly Durrell Wildlife Park; Channel Islands), and ZSL London Zoo (UK). We collected behavioral and physiological (i.e., hormone levels) data from June 2022 to February 2023. Despite this lemur species exhibiting a seasonal pattern of breeding in the wild, there is no clear breeding season in captivity with births occurring all over the year (Beattie and Feistner 1998; ZIMS 2024) (Table 1). All male‐female pairs were housed in natural outdoor enclosures with access to indoor areas maintained at 25°C–28°C during the cold season. The lemurs had the opportunity to remain in constant full contact and could avoid being seen by visitors if they chose. No other species were present in the enclosures, and no additional enrichment was provided to the subjects during data collection.

**Table 1:** Study subjects and sampling periods.

Zoo	Name	Sex	Age at study start (yrs)	Period of sampling
*Birmingham Zoo*	Zoma	Male	15	27/06/22 – 10/08/22
	Bozy	Female	12	
*Mulhouse Zoo*	Kwic	Male	8	25/07/22 – 26/08/22
	Manon	Female	12	
*Jersey Zoo*	Brian	Male	13	19/09/22 – 21/10/22
	Bala	Female	17	
*London Zoo*	Rocky	Male	15	09/01/23 – 10/02/23
	Hazo	Female	3	

### 2.2 Study Protocol

We divided the study period into three phases: pre‐ enrichment (i.e., before enrichment: 10 days); enrichment (i.e., during enrichment: 6 days); post‐ enrichment (after enrichment: 10 days). We carried out behavioral observations and fecal sampling every study day (6 days per week) between 8AM and 1PM (5 h per day), as the lemurs are more active in the morning (Haring and Davis 1998), excluding Sundays to avoid the peak visitor day. We assessed the effects of the enrichment combining the observation of sexual behaviors and behavioral indicators of welfare (e.g., olfactory behaviors, aggressive, abnormal and self‐directed behaviors; Nielsen et al. 2015; Papageorgiou and Simitzis 2022; Truelove et al. 2020) and fecal endocrinology (e.g., fecal cortisol levels in both males and females as well as testosterone levels in males).

### 2.3 Odour Sampling and Analyses

As previously described by Fontani et al. (2022), we used positive reinforcement training (Spiezio et al. 2015) for 5 days to train a breeding female Alaotran gentle lemur at the Jersey Zoo to allow us to obtain anogenital odour secretions cooperatively. At the time of data collection, the female was 7 years old and had bred regularly over the previous 3 years. We then collected anogenital odour samples (*n* = 35 samples) every morning before feeding (8‐8:30AM) by rubbing a sterile cotton swab 10 times around the wall of the vulva and using steady pressure. Moreover, we exposed control swabs to the air once a week to identify any compounds that did not derive from the lemurs. We placed all samples and controls into sterile vials and immediately stored them in a −20°C freezer at the zoo. We then transferred the vials to the Rosalind Franklin Science Centre, University of Wolverhampton, using a freezer box with ice packs to avoid any risk of defrosting, for laboratory analyses.

To define the oestrus cycle, we used the patterns of fecal progesterone metabolites and 17β‐estradiol levels to determine the occurrence of ovulatory windows and the timing of fertility in the breeding female (we considered the time lag between steroid secretion and excretion in feces).

We conducted the hormone analyses at the Rosalind Franklin Science Centre, University of Wolverhampton. The protocols for hormone sampling and measurement are detailed in Fontani et al. (2022). In summary, we collected 54 fecal samples from the breeding female and immediately stored them at −20°C in the zoo's freezer. We then transferred the samples to the University of Wolverhampton using a freezer box with ice packs to prevent thawing. In the laboratory, we lyophilized the fecal samples for 72 h, pulverized them, and sieved them. We extracted 0.05–0.1 g of the fecal powder in 3 ml of 80% methanol in a 15 ml plastic tube. We vortexed the solution for 15 min with a multi‐tube vortexer, then centrifuged it for 20 min at 3266 × *g*, and immediately stored the supernatant at −20°C.

We assessed progesterone metabolites and 17β‐estradiol levels using ELISA kits (DetectX Progesterone Metabolites K068‐H5 and DetectX 17β‐Estradiol K030‐H5, Arbor Assays, USA). We prepared the samples by diluting them 1:10 with the assay buffer and followed the kit protocols for all assays. Each fecal sample and standard were tested in duplicate. We processed the data using a four‐parameter logistic (4PL) fitting programme (MyAssays, Brighton, UK), and reported the concentrations as pg/mg. The mean intra‐assay coefficient of variation, derived from four samples tested with eight replicates on a single plate, was 10.2% for progesterone and 7.6% for estradiol. The mean inter‐assay coefficient of variation, calculated from four quality control samples measured in duplicate across three plates, was 12.3% for progesterone and 8% for estradiol.

We estimated the ovulatory window as the period when estradiol levels increased while progesterone levels initially decreased followed by a constant rise in progesterone for at least 5 days.

We then investigated the volatile component of odour signals using solid‐phase microextraction (SPME) and gas chromatography‐mass spectrometry (GC‐MS) techniques. Briefly, we introduced a 65 µm polydimethylsiloxane/divinylbenzene SPME syringe needle through the vial septum and exposed the fibre to the headspace above the sample in the vial for 15 min at 40°C. We analysed the adsorbed volatile analytes of all samples using a 5975 C mass spectrometer (Agilent Technologies) EI, 70 eV, coupled directly to a 7890B gas chromatograph (Agilent Technologies) equipped with a fused silica HP5‐MS UI capillary column (Agilent Technologies) 30 m × 0.25 mm cross‐bonded 5%‐phenyl‐95% dimethylpolysiloxane, film thickness 0.25 µm. We maintained the injector and transfer line temperatures at 270°C and 280°C, respectively. We made injections in split‐less mode (purge valve opened after 1 min) with a constant flow of helium carrier gas of 1 ml per min. We started the oven temperature programme at 45°C for 2 min, then raised it by 4°C per min to 170°C, and finally by 20°C per min to 300°C 40.

We assessed possible environmental contamination via blank analyses using an empty 10 mL vial (Supelco) and control swabs following the same procedure as for the samples and conditioned the fibre at 260°C pre‐injection for 5 min and 260°C postinjection for 20 min to avoid any possible carry‐over effects. We analysed all samples within 1 week to minimize inter‐assay variability. We overlaid chemical profiles from control swabs on animal chemical profiles to identify compounds that did not derive from the animals and removed these from the swab results.

We first tentatively identified eluted compounds by comparing the experimental spectra with those of the mass‐spectral library in ChemStation (Agilent Technologies) and NIST Database (National Institute of Standards and Technology), version MSD F.01.01.2317 (Agilent Technologies). We accepted a putative identification if the minimum matching factor was higher than 90%. After that, we carried out the unequivocal identification of the key compounds distinguishing the fertile window of the breeding female using the same swabs and vials as for lemur sample collection and then comparing these compounds with standard compounds injected and analysed by applying the same SPME and GC‐MS protocol (Walker and Vaglio 2021).

**Figure 1:**
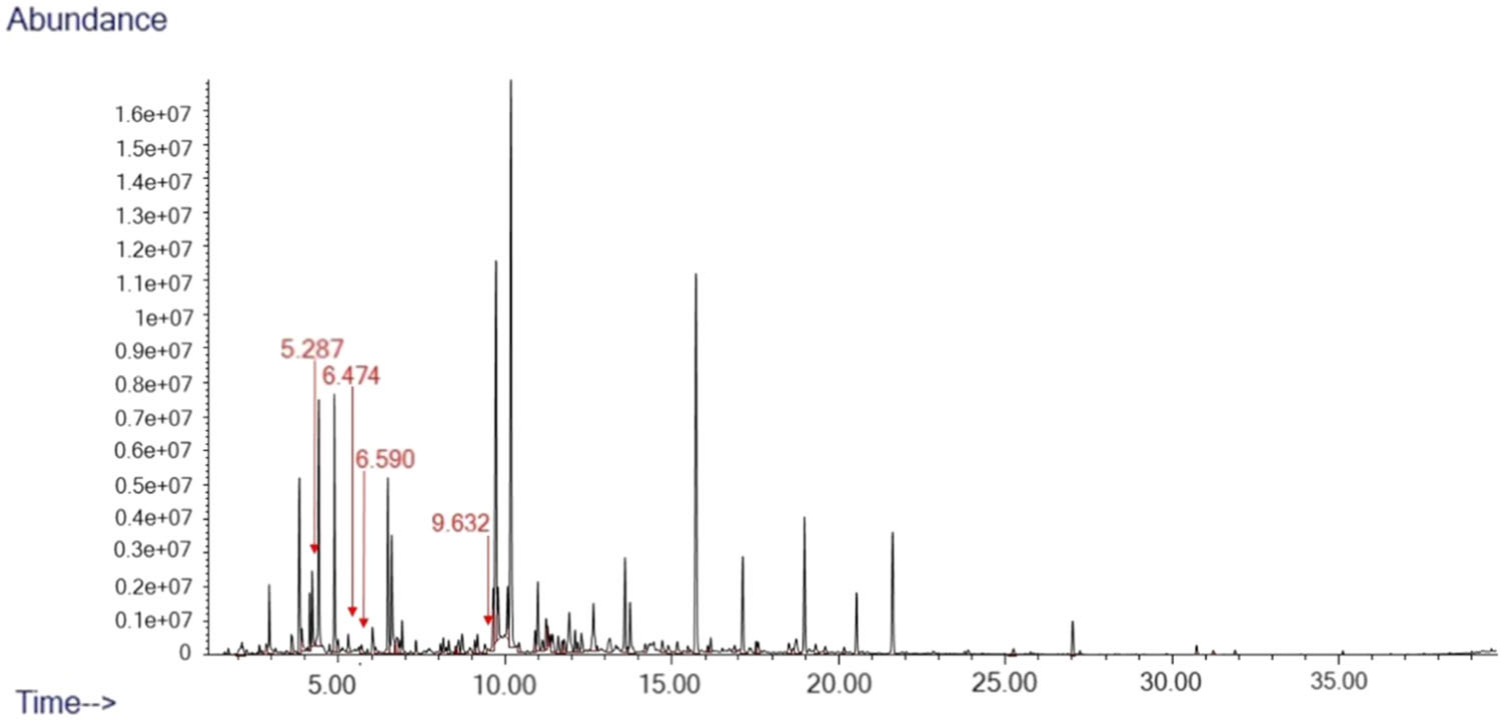
Example chromatogram from female gentle lemur, anogenital odour sample from fertile period. Four key compounds are pointed out with a red arrow: 4‐Methyl 3‐Hexanone (RT 5.287), 3‐Heptanone (RT 6.474), 2‐Heptanone (RT 6.590), 3‐Octanone (RT 9.632).

As reported by our previous study (Fontani et al. 2022), we identified a total of 78 distinct peaks in 35 swab samples of Aloatran gentle lemur anogenital secretions which were not present in the control swabs (Figure 1). Specifically, four compounds (2‐heptanone; 3‐heptanone; 3‐octanone; 4‐methyl, 3‐hexanone) distinguished the chemical profile of odour secretions during the lemur ovulation window.

### 2.4 Scent Enrichment

We prepared standard dilutions using similar methods to other authors who identified volatile organic compounds in owl monkeys (*Aotus spp*.) (Spence‐Aizenberg et al. 2018). Briefly, we diluted each chemical compound (2‐heptanone; 3‐heptanone; 3‐octanone; 4‐methyl, 3‐hexanone) separately, placing 1.5 mL of HPLC grade methanol in 15 mL test tube, adding 5 µL of compound and 3.5 mL of de‐ionized water, and then we vortexed for 15 s to dissolve the compound in the mixture. We compared both the retention times of key compounds and standards and the overall patterns of the mass spectra. We accepted the identification only if both the parameters were satisfied. 2‐heptanone, 3‐heptanone and 3‐octanone were commercially available (Agilent), while 4‐methyl, 3‐hexanone was synthesized in our chemistry laboratory. Once the identification was certain, we added 1 mL of each diluted compound into a new test tube and vortexed for 30 s to produce the scent mixtures to test as olfactory enrichment.

**Figure 2:**
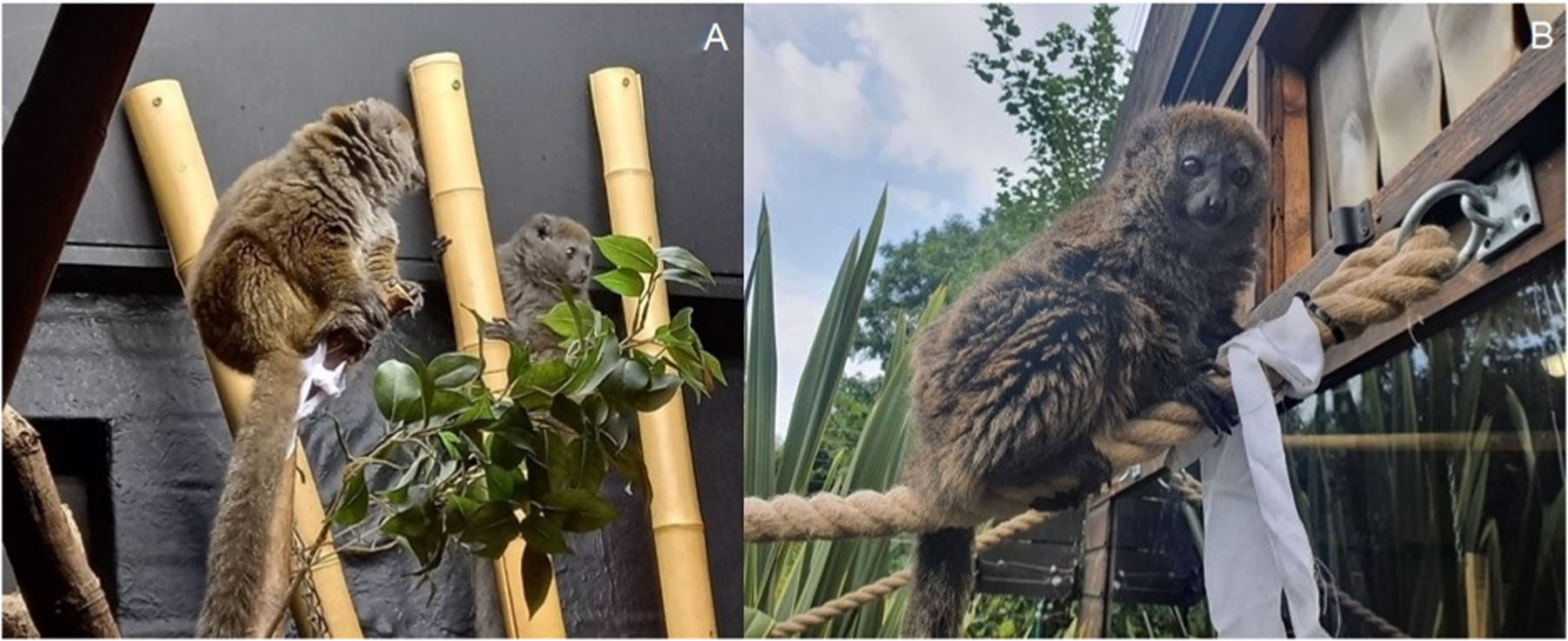
Gentle lemurs interacting with scent enrichment in the indoor enclosure at ZSL London Zoo. Photo by Anna Beatrice Costantini (A). Male gentle lemur interacting with scent enrichment in the outdoor enclosure at Birmingham Wildlife Conservation Park. Photo by Georgia Callagan (B).

We presented the enrichment to the study subjects applying the same protocol as in our prior study (Vaglio et al. 2021). Briefly, we used white cotton sheets cut into 75 cm long and 5 cm wide strips, which were soaked with 20 drops of scent mixture diluted with 12 mL of cold boiled water. Newly soaked cotton strips were prepared each enrichment day. We placed 2 unscented (controls) and 6 scented strips on the climbing frames both indoor and outdoor (Figure 2A,B) and removed them at the end of the period of observations every day. To avoid habituation, we randomized the locations of both scented and unscented cotton strips daily.

### 2.5 Behavioral Data Collection

We collected behavioral data using all occurrences of some behaviors sampling method (Altmann 1974), focusing on aggressive, abnormal, self‐directed, olfactory, and sexual behaviors (Table 2). We also used ad libitum sampling method (Altmann 1974) to collect environmental and contextual data (e.g., weather conditions, changes in the enclosure's surroundings). We recorded a total of 515 h of observations over the study period.

**Table 2:** Ethogram of selected behaviors for the study subjects modified from (Fontani et al. 2022).

Behaviors	Description
*Olfactory behaviors*	
Brachial scent marking	Scratching object with lower dentition, then rubbing spot on brachial glands (on arms). Only males.
Anogenital scent marking	Rubbing object with genitalia, then sit‐rubbing repeatedly whilst depositing urine.
Territorial marking	Grabbing, biting, chasing, lunging, confrontation display to conspecifics.
Tail‐scent marking	Standing on hind legs with tail bent towards them, rubbing object on insides of wrists and tail simultaneously.
Sniff genitals	Place the nose less than 3 cm from the anogenital area of a conspecific and lick it.
Sniff substrate	Inspecting, sniffing, touching, biting, licking a substrate for at least 2 s.
*Sexual behaviors*	
Anogenital self‐grooming	Grooming of genital area, using fingers or mouth.
Follow	Male approaches female from behind and follows closely (less than 1 m).
Penile erection	The subject shows a conspicuously erect red penis.
Mating calls	Female produces distinct singly or in series call, while soliciting copulation and during mating.
Attempt mounting	Male approaches female, clasps, orients body for copulation; female chatters at and/or cuffs the male, male releases female.
Copulation	Male approaches, female crouch, male introduces sperm into the female's reproductive tract.
*Aggressive behaviors*	
Intimidation	The subject emits a short vocalization toward a conspecific to warn it not to come closer.
Chase	The subject chases a conspecific; chasing him on the ground or scrambling to reach him.
Bite	The subject bites a conspecific.
*Self‐directed behaviors*	
Self‐scratching	The subject rubs its own body at a fast pace.
*Abnormal behaviors*	
Pacing	The subject walks back and forth in a distinct, unchanged pattern though the enclosure.

We tested interobserver reliability by measuring the degree of agreement in behavior identification between two observers (A.B.C. & G.C.) at the host zoos (Wark, Wierzal, and Cronin 2021). Specifically, we used Cohen's Kappa coefficient to measure the agreement between the observers and obtained 83%, which is considered “almost perfect” (Viera and Garrett 2005).

### 2.6 Fecal Hormone Sampling and Measurements

We collected a total of 180 fecal samples over the study period (Table 3). We collected the samples every morning during the behavioral observations, right after defecation was observed, when the identity of the study subject was certain. As diurnal secretion patterns of hormones, such as cortisol and testosterone, may be detected in fecal samples (especially for small‐bodied species), we restricted the sampling period to approximately the same time of the day (Hodges and Heistermann 2011). We stored the samples in a − 20°C freezer on site immediately after collection. At the end of the study period, we transferred the samples to the Rosalind Franklin Science Centre – University of Wolverhampton using a cold bag with ice packs to avoid any risk of defrosting.

**Table 3:** Observation time, counts of behaviors and number of fecal samples collected for males and females in the three phases of experiment in the four zoos.

Zoo	Individual	Enrichment phase	Olfactory behaviors (count)	Sexual behaviors (count)	Aggressive behaviors (count)	Abnormal and self‐directed behaviors (count)	Observation time (min)	Fecal samples (number)
**Birmingham**	*Bozy (F)*	Pre	48	8	0	0	3000	8
		Enr	34	21	0	0	1800	7
		Post	124	17	0	0	2700	8
	*Zoma (M)*	Pre	71	8	0	0	3000	9
		Enr	51	12	2	0	1800	6
		Post	148	14	0	0	2700	6
**Mulhouse**	*Manon (F)*	Pre	69	10	4	2	3000	10
		Enr	27	5	5	6	1800	7
		Post	50	3	8	13	3000	10
	*Kwic (M)*	Pre	285	14	0	0	3000	11
		Enr	134	11	1	0	1800	7
		Post	197	18	1	0	3000	10
**Jersey**	*Bala (F)*	Pre	35	3	1	0	3000	7
		Enr	68	3	0	0	1800	7
		Post	81	7	3	0	3000	7
	*Brian (M)*	Pre	275	3	1	0	3000	10
		Enr	180	24	1	0	1800	7
		Post	268	21	2	0	3000	9
**London**	*Hazo (F)*	Pre	50	18	2	0	1641	7
		Enr	59	11	0	0	619	4
		Post	32	23	0	0	1164	5
	*Rocky (M)*	Pre	44	0	0	0	1337	8
		Enr	22	0	0	0	574	4
		Post	48	2	0	0	925	6

Abbreviations: Enr, enrichment; F, female; M, male; Pre, pre‐enrichment; Post, post‐enrichment.

#### 2.6.1 Hormone Analyses

We used a freeze‐drying machine (Christ R, Beta 1–8 LSC plus, Osterode am Harz, Germany) to lyophilize the fecal samples for 72 h, and then we pulverized them using a pestle and mortar. We sieved the fecal powder through a stainless‐steel strainer, aperture 250 mic, to separate the fecal residue from any fibrous material. With regard to extraction, we followed the methods of our prior studies (Fontani et al. 2022; Elwell, Fontani, and Vaglio 2024). Briefly, we extracted 0.05–0.1 g of fecal powder in 3 mL of 80% methanol using a 15 ml plastic tube and vortexing it for 15 min with a multi‐tube vortexer (Grant Instruments R, Multi‐Vortexer V‐32, Cambridge, UK). Right after centrifugation for 20 min at 3,266 xg, we stored the supernatant at −20°C.

When analysing fecal hormones, we considered the time course of hormones metabolite excretion relative to the production and circulation of the native hormones (Hodges and Heistermann 2011; Wheeler et al. 2013). We measured fecal cortisol and testosterone levels using commercially available enzyme‐linked immunosorbent assay (ELISA) kits (Enzo Life Sciences Cortisol, ADI‐900‐071, New York, USA and DetectX Testosterone K032‐H5W, Arbor Assays R, USA) following kits instructions. Before analysis, we diluted all the samples 1:1 with the assay buffer provided by the kits. We assayed all standards and fecal samples in duplicates, with samples showing a coefficient of variation (CV) exceeding 15% being re‐analysed (Macagno et al. 2020). All samples were randomly distributed on the assay plates. We analysed assay data applying a 4‐parameter logistic fitting programme (MyAssays R, Brighton, UK). Concentrations were expressed as pg/mg. Mean intra‐assay coefficient of variation for cortisol, tested on four quality control samples (two males and two females) with eight replicates within a single assay plate, was 7.77% ± 1.27%, while for testosterone, tested on three control samples (all males), was 9.35% ± 2.57%. Mean inter‐assay coefficient of variation, tested on the same samples measured with four replicates across three assay plates, was 15.04% ± 5.21% for cortisol and 5.96% ± 1.42% for testosterone.

### 2.7 Statistical Methods

#### 2.7.1 Behavioral Analyses

We conducted all analyses in R, version 4.3.0 (R Core Team 2023). To explore the effects of scent enrichment on subject behavior, we fitted two negative binomial generalized linear mixed models (GLMMs) using the glmer. nb() function from the lme4 package (Bates et al. 2018). We focused on olfactory and sexual behaviors, excluding aggressive, abnormal and self‐directed behaviors due to their infrequent occurrence. For each model, we set the count of olfactory or sexual behaviors recorded per observation period as the dependent variable. We entered subject sex (male/female), enrichment phase (pre‐, during, and post‐ enrichment), and their interaction as fixed effects. We also included Zoo as a fixed effect to account for inter‐zoo differences that might influence the subjects' behavior. To address unequal observation times across individuals and treatment conditions, we incorporated observation time (in minutes) as an offset in both models. We included subject identity (i.e., ‘ID’) as a random effect. Initially, we specified models with both random intercepts and random slopes for enrichment phase by subject ID, but this approach led to singular fits. To resolve this, we began with our ideal model and removed terms necessary to achieve a non‐singular fit (Barr et al. 2013). Removing random slopes resolved the issue, resulting in final models with subject ID as a random intercept only.

#### 2.7.2 Hormone Analyses

We explored the effects of enrichment phase (pre‐, during, and post‐enrichment) on fecal cortisol concentration by fitting a Linear Mixed Model (LMM) with the lmer() function from the lme4 package (Bates et al. 2018). We used log‐transformed (base 10) cortisol concentration as the dependent variable to meet the assumption of normally distributed residuals. The model included zoo, enrichment phase, subject sex, and the interaction between enrichment phase and subject sex as fixed effects. We included subject identity (ID) as a random effect. Initially, we specified random intercepts and slopes for enrichment phase by subject ID, but this approach resulted in a singular fit. To resolve this, we simplified the model, retaining only random intercepts for subject ID.

Finally, we fitted a fourth model for testosterone concentration. Because we measured testosterone only in male fecal samples, we included enrichment phase and subject ID as fixed effects, excluding subject sex. As there were only four subjects (each zoo had only one male), we included subject ID as a fixed effect and not as a random effect. We fitted this model as a linear regression model (LM) using the lm() function from the stats package (R Core Team, 2013). We removed two unusually high testosterone values from the data set, as these were inconsistent with known natural fluctuations of testosterone levels.

#### 2.7.3 Model Interpretation, Assumptions, and Diagnostics

For our models, we set “pre‐enrichment” as the reference category for enrichment phase, “female” as the reference category for sex, and “Birmingham” as the reference category for zoo. To assess the effects of interaction terms in our models (Olfactory, sexual, and cortisol models), we used the ANOVA() function to run likelihood ratio tests that compared the full models to reduced versions without interactions. We also compared each model, including the testosterone model, to its respective null model using the ANOVA() function. We assessed the significance of independent variables in the final models using the drop1() function (R Core Team 2023). Where appropriate, we employed the emmeans package (Lenth et al. 2019) to calculate marginal means and perform post hoc comparisons across the three treatment conditions (pre‐, during, and post‐ enrichment phases). To adjust for multiple comparisons, we applied the Tukey method with an alpha level of 0.05. We used the sjPlot package for model visualization.

We performed tests for over dispersion and zero inflation using the DHARMA package (Hartig 2020). We used the vif() function within the car package (Fox and Weisberg 2019) to derive variance inflation factors (VIF) from standard linear models with no random effects and included all variables separately with no interactions to rule out collinearity (Cassidy et al. 2020). We found no issues with collinearity in our models (maximum VIF for all models = 1.01). We assessed model stability by excluding each study subject individually in models and we then compared these model estimate subsets with that of the models produced using the full data set (Cassidy et al. 2020).

## 3 Results

### 3.1 Behavioral Results

Over the course of the study period, we collected 515 h of observational data across the four zoos. A breakdown of observation time per subject, per enrichment phase (pre‐, during, and post‐enrichment), including counts of olfactory and sexual behaviors is outlined in Table 3 and summarized as follows: total observation time (minutes)—males: 25,936, females: 26,524; olfactory behaviors (count)—males: 1,723, females: 677; sexual behaviors (count)—males: 127, females: 129. While subjects in the other observed groups were consistently visible, the layout of the enclosure at London Zoo occasionally hindered visibility to the observer, despite the equal observation duration.

### 3.2 Olfactory Behaviors

**Figure 3:**
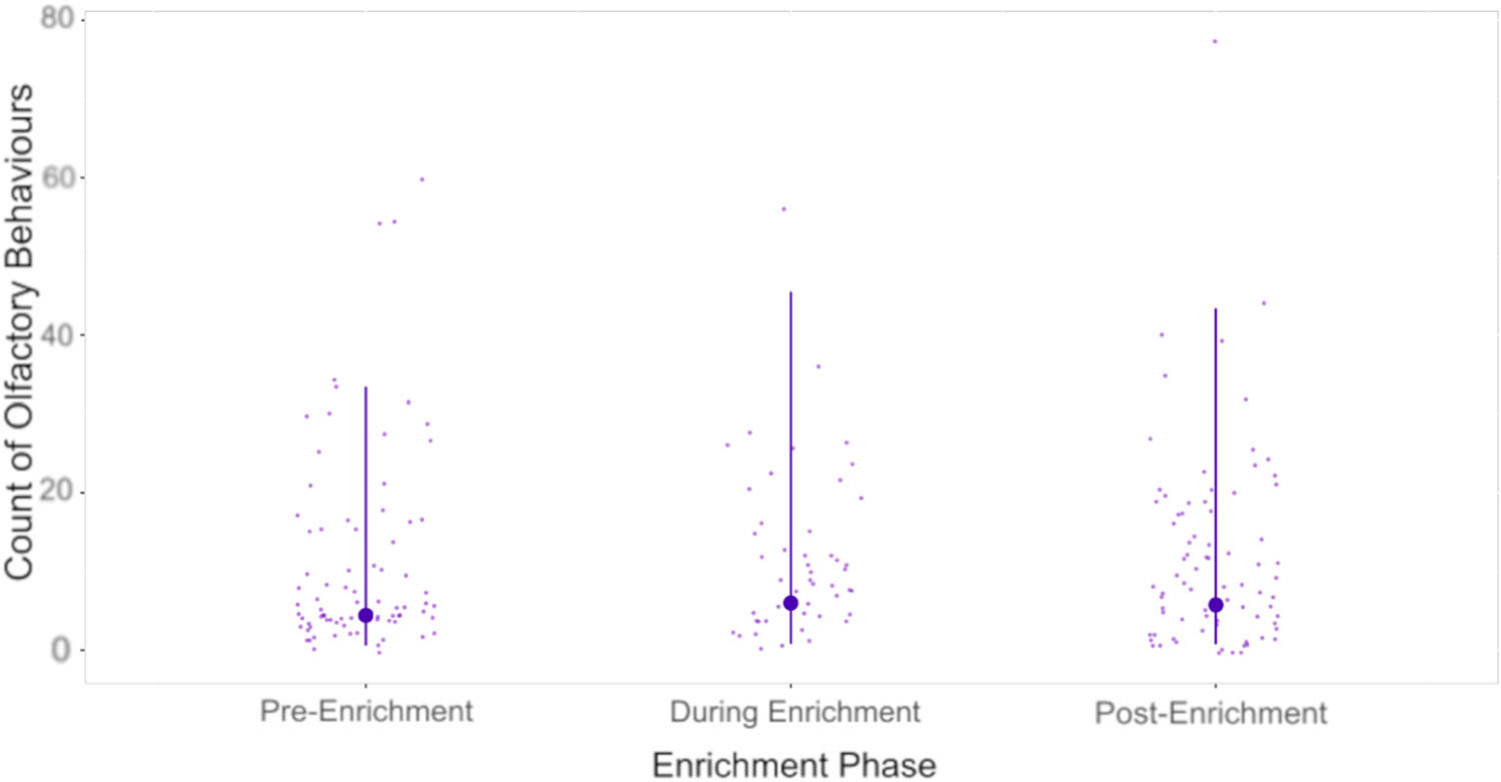
Predicted count of olfactory behaviors in the pre‐, during, and post‐enrichment phase of the experiment. Estimates are back transformed to the response level (counts per observation period). Smaller, lighter points represent actual data points collected during the study. Larger darker points connected to error bars represent model estimates and 95% confidence intervals respectively.

The reduced model, which included the three main effects but not the interaction term, provided a significantly better fit to the data than the null model (χ² = 15.05, df = 6, *p* = 0.02). Adding the interaction term did not significantly improve the fit (χ² = 4.35, df = 2, *p* = 0.11), indicating that the data do not support an interaction between the subject sex and enrichment phase. Consequently, all subsequent analyses and interpretations are based on the reduced model. Likelihood ratio tests revealed significant effects of both sex and enrichment phase on the rate of olfactory behaviors (sex, LRT = 7.50, df = 8, *p* = 0.006; enrichment phase, LRT = 6.99, df = 7, *p* = 0.03). In contrast, the variable ‘zoo’ did not significantly affect the rate of olfactory behaviors (LRT = 1.80, df = 6, *p* = 0.61). Estimates for the reduced model are presented in Table 4 and in the appendix (Table A1). After adjusting for multiple comparisons using the Tukey method, our post hoc analyses did not find significant pairwise differences between any of the enrichment phases (Figure 3, Table 5). This lack of significant differences in our post hoc analyses given the significant effect in our model may indicate a small effect size or insufficient power in the analysis. The fitted model did not deviate significantly from the assumptions of overdispersion or zero inflation (dispersion = 0.94, *p*‐value = 0.86, ratio Obs/Sim zeros = 0.92, *p*‐value = 0.992).

**Table 4:** Mixed model analyses showing the effect of enrichment (pre‐, during, and post‐ enrichment) on the number of olfactory and sexual behaviors.

Analysis	Dependent	Predictors	Estimate	Std. error	z value	*p*‐value
**Negative binomial GLMM**	*Count of Olfactory Behaviors*	Intercept	−4.06	0.26	−15.38	< 0.001
		Enrichment: During	0.30	0.14	2.25	0.024
		Enrichment: Post	0.26	0.12	2.25	0.025
		Sex: Male	0.80	0.23	3.56	< 0.001
		Zoo: Jersey	0.41	0.32	1.29	0.197
		Zoo: London	0.38	0.32	1.17	0.241
		Zoo: Mulhouse	0.26	0.32	0.81	0.421
**Negative binomial GLMM**	*Sexual Behaviors*	Intercept	−5.59	0.55	−10.13	< 0.001
		Enrichment: During	0.88	0.19	4.73	< 0.001
		Enrichment: Post	0.61	0.18	3.49	< 0.001
		Sex: Male	−0.33	0.50	−0.66	0.511
		Zoo: Jersey	−0.58	0.69	−0.85	0.396
		Zoo: London	−0.08	0.72	−0.11	0.914
		Zoo: Mulhouse	−0.60	0.68	−0.87	0.385

*Note:* All models use “pre‐ enrichment” phase, “female” and “Birmingham Zoo” as the reference intercept. *p*‐values reported in the table represent those produced by the default summary() function, and do not reflect the p‐values from the likelihood ratio tests reported in text.

**Table 5:** Pairwise contrasts of the rate of olfactory behaviors by male and female subjects across the three experimental phases (pre‐, during, and post‐ enrichment).

Contrast	Ratio	SE	*z*‐ratio	*p*‐value
*Pre ‐ During*	0.74	0.10	−2.25	0.063
*Pre ‐ Post*	0.77	0.09	−2.25	0.063
*During ‐ Post*	1.04	0.14	0.30	0.952

### 3.3 Sexual Behaviors

**Figure 4:**
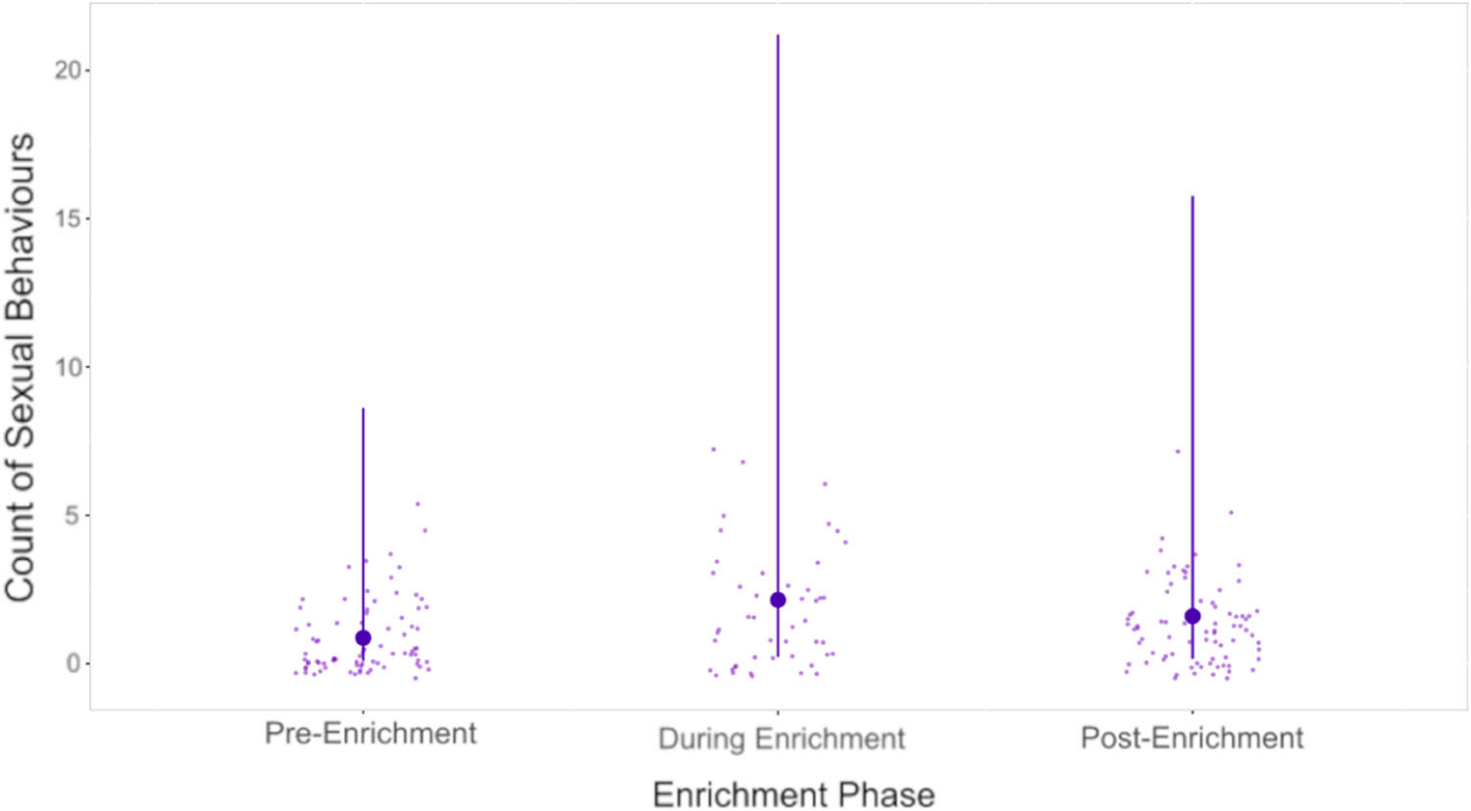
Predicted count of sexual behaviors in the pre‐, during, and post‐ enrichment phases of the experiment. Estimates are back transformed to the response level (counts per observation period). Smaller, lighter points represent actual data points collected during the study. Larger, darker points connected to error bars represent model estimates and 95% confidence intervals respectively.

The reduced model, which included the three main effects but excluded any interaction terms, fit the data significantly better than the null model (χ² = 24.01, df = 6, *p* = 0.001). Adding the interaction term to the model did not significantly improve the fit (χ² = 1.83, df = 2, *p* = 0.40), indicating that the data do not support an interaction effect between subject sex and enrichment phase. Based on this result, we selected the reduced model for further analysis and interpretation. Likelihood ratio tests revealed a significant effect of enrichment phase on sexual behaviors (LRT = 26.17, df = 7, *p* < 0.001). However, neither subject sex nor zoo had a significant effect (sex, LRT = 0.11, df = 8, *p* = 0.74; zoo, LTR = 0.49, df = 6, *p* = 0.92). Estimates for the reduced model are presented in Table 4 and in the appendix (Table A2). Pairwise comparisons between enrichment phases revealed a significant increase in the rate of sexual behaviors when moving from the pre‐ enrichment phase to both the enrichment and post‐ enrichment phases (pre‐during, *p* < 0.001; pre‐post, *p* = 0.001; see Figure 4, Table 6). The final reduced model did not deviate significantly from the assumptions of overdispersion or zero inflation (dispersion = 0.56, *p*‐value = 0.864, ratio Obs/Sim zeros = 1.01, *p*‐value = 0.968).

**Table 6:** Pairwise contrasts of the rate of sexual behaviors by male and female subjects across the three experimental phases (pre‐, during, and post‐ enrichment)

Contrast	Ratio	SE	*z*‐ratio	*p*‐value
**Pre ‐ During**	**0.41**	**0.07**	**−4.92**	< 0.001
**Pre ‐ Post**	**0.54**	**0.09**	**−3.50**	0.001
During ‐ Post	1.34	0.22	1.78	0.176

*Note:* Significant differences across enrichment phases are indicated in bold.

### 3.4 Aggressive and Abnormal and Self‐Directed Behaviors

Aggressive, abnormal and self‐directed behaviors were excluded from the statistical analysis due to their infrequent occurrence over the total 515 h of observation. Specifically, we documented a total of 30 aggressive behaviors, predominantly exhibited by the female at the Mulhouse Zoo, particularly during feeding. We recorded a total of 21 instances of abnormal and self‐directed behaviors (pacing), all displayed by the female at the Mulhouse Zoo, consistently preceding feeding. A comprehensive breakdown of the frequency of the different behaviors within each behavioral group is presented in Table 3.

### 3.5 Hormonal Analyses

The reduced cortisol model, which included the three main effects but excluded their interaction, fit the data significantly better than the null model (χ² = 25.52, df = 6, *p* < 0.001). Including the interaction term did not improve the model's fit relative to the reduced version (χ² = 1.75, df = X2, *p* = 0.42). The reduced model revealed a significant effect of zoo on cortisol concentration (F test: F = 20.17, df = 3, 2.18, *p* = 0.01). In contrast, neither enrichment phase (F = 0.53, df = 2, 170.39, *p* = 0.59) nor sex (F = 1.22, df = 1, 3.20, *p* = 0.35) had a significant effect. We used a Shapiro‐Wilk test to confirm that the residuals followed a normal distribution (W = 0.99, *p* = 0.178). The testosterone model also fit the data significantly better than the null model (F = 22.09, df = 5, *p* < 0.001). Similar to the cortisol model, our testosterone model found a significant effect of zoo on fecal testosterone levels (F test: χ² = 3444.2, df = 3, *p* < 0.001), but no significant effect of enrichment phase (χ² = 27.8, df = 2, *p* = 0.61). As with the cortisol model, we ran a Shapiro Wilk test to test the assumption that the residuals were normally distributed (W = 0.98, *p* = 0.073). Estimates for the reduced model are presented in Table 7. Full model outputs are presented in Tables A3 and A4.

**Table 7:** Mixed model analyses showing the effect of enrichment phase (pre‐, during, and post‐ treatment) on log cortisol and testosterone concentrations

Analysis	Dependant	Predictors	Estimate	Std. error	df	*t* value	*p*‐value
**LMM**	*Log Cortisol concentration in fecal samples*	Intercept	4.48	0.10	4.08	45.04	< 0.001
		Enrichment: During	−0.01	0.06	170.47	−0.12	0.904
		Enrichment: Post	−0.06	0.06	170.43	−0.97	0.334
		Sex: Male	−0.09	0.08	3.20	−1.11	0.345
		Zoo: Jersey	−0.75	0.12	3.15	–6.34	0.006
		Zoo: London	−0.10	0.12	3.60	−0.86	0.443
		Zoo: Mulhouse	−0.64	0.12	2.99	−5.53	0.012
**LM**	*Testosterone concentration in fecal samples (male subjects only)*	Intercept	34.59	1.43		24.18	< 0.001
		Enrichment: During	−1.01	1.45		−0.70	0.489
		Enrichment: Post	−1.19	1.33		−0.90	0.371
		Zoo: Jersey	−16.55	1.61		‐10.26	< 0.001
		Zoo: London	−8.25	1.77		−4.66	< 0.001
		Zoo: Mulhouse	−12.53	1.59		−7.89	< 0.001

*Note:* All models use “pre‐ enrichment” phase, “female” and “Birmingham Zoo” as the reference intercept. P‐values reported in the table represent those produced by the default summary() function, and do not reflect the *p*‐values from the F tests reported in text.

## 4 Discussion

Studies of olfactory enrichment are less frequent than those of other types of enrichment, particularly with regard to nonhuman primate species (Elwell and Vaglio 2023), and their findings are contradictory (Baker, Taylor, and Montrose 2018; Gronqvist et al. 2013). Nevertheless, recent evidence suggests that more attention should be paid to species‐specific biologically relevant scents as they might be able to influence both welfare status and mating behaviors in captive populations (reviewed in Elwell and Vaglio 2023). The effectiveness of scent enrichment programmes depends on the target species (Carlstead, Seidensticker, and Baldwin 1991), with differences having also been found between sexes (Clark, Melfi, and Mitchell 2005), as males and females may respond differently towards conspecific scents due to reproductive and mate quality cues (Boulet et al. 2010). In this research work, we designed, tested, and evaluated the effects on welfare indicators and sexual behavior of a novel scent enrichment, via behavioral and endocrinological approaches. Specifically, we studied four unrelated groups of captive Aloatran gentle lemurs adequately housed in modern zoo enclosures with comparable environment and diet.

In contrast to our prediction, we did not find a significant increase in rates of olfactory behaviors during the enrichment phase. However, our prior analysis of scented versus unscented strips (Costantini et al. 2024) demonstrated that both sexes exhibited a significant increase in olfactory behaviors and proximity towards the scented cotton strips, indicating that olfactory stimuli could play a valuable role in enriching the captive environment. Our finding in the present study is consistent with results from studies on other primate species (e.g., gorillas, *Gorilla gorilla gorilla*: Wells et al. 2007; ring‐tailed lemurs, *Lemur catta:* Baker, Taylor, and Montrose 2018) which also showed no significant effects of scent enrichment programmes on the rates of olfactory behaviors. Gronqvist et al. 2013, on the other hand, found a significant increase in olfactory behaviors in captive Javan gibbons (*Hylobates moloch*), but they did find that interest in the new olfactory stimulus decreased rapidly after the first day of treatment. These studies focused on essential oils and not biologically or ecologically relevant scents, which they may have contributed to the lack of effects on olfactory behaviors. Thus, this could suggest that key compounds were missing from our newly developed scent enrichment and so it did not provide a biologically‐relevant scent signal. Additionally, as the compounds used for the enrichment were volatile, they may not have persisted in the enclosures for much time which could have limited the frequency of olfactory behaviors observed. Thus, the delivery method of the enrichment may need to be reconsidered in relation to the properties of the compounds used.

We found a significant increase in sexual behaviors, performed by both sexes, during the enrichment phase. Specifically, we observed anogenital self‐grooming and erection behaviors to increase, which are also considered sexual behaviors in several other mammal species (e.g., male and female rats, *Rattus norvegicus*: Moore 1986; male rodents: Hull and Dominguez 2007). Our findings are consistent with those in rhesus monkeys (Ruiz de Elvira, Herndon, and Wilson 1982) where, after introducing oestrogen‐treated females into an established group, both males and females showed increased sexual behaviors. On the other hand, contrary to what we predicted, we did not observe any occurrence of mating behavior. The lack of mating behavior in our study could be due to mating taking place outside of the daily observational times, or to the fact that the breeding status of our study subjects was uncertain at the time of observation. Nevertheless, our study suggests biologically relevant scent enrichments may have the potential to increase sexual behaviors in captive animals.

We did not find any significant decrease in cortisol levels after the enrichment phase, which may be due to several factors. For instance, cortisol is commonly used to measure both acute (Volfová et al. 2019) and chronic stress (Sapolsky 2002) levels. Furthermore it is often related to group instability (Preis et al. 2019; Vandeleest et al. 2019), aggressive patterns (Muller and Wrangham 2004; Yamanashi et al. 2016) and displacement activities (Maestripieri et al. 1992) in primate species. As we observed stress‐related behaviors rarely during the study period, we assumed that study subjects had good welfare status and, therefore, the potential for impact by the scent enrichment was limited. Prior work on rhesus monkeys showed that, although environmental enrichment may lead to behavioral improvements, it does not always affect adrenal function (Schapiro et al. 1993). Also, a study on a single‐housed orangutan (*Pongo pygmaeus*) highlighted that cortisol metabolites significantly decreased over the long period to lower levels than those found during the pre‐ enrichment phase, which could indicate that long‐term data collection after the enrichment phase could have led to different endocrinology results (Schilbach Pizzutto et al. 2008).

We did not find any significant change in male testosterone levels, contrary to what we expected in response to exposure to female fertile odour. Prior studies showed that in other primate species (e.g., stump‐tailed macaques, *M. arctoides*: Cerda‐Molina et al. 2006; common marmosets, *Callithrix jacchus*: Ziegler et al. 2005; humans: Miller and Maner 2010), males exposed to the odour of an ovulating female display higher testosterone levels in comparison with males exposed to the scent of a non‐ovulating female or a control scent. Moreover, the interaction with oestrogen‐treated females increased testosterone levels in male rhesus monkeys even outside the breeding season (Ruiz de Elvira, Herndon, and Wilson 1982). However, these primate species are non‐lemur/non‐strepsirrhine species, with strepsirrhine primates exhibiting considerably different reproductive physiologies from haplorrhine species. According to the “challenge hypothesis” (Wingfield et al. 1990), in multi‐male multi‐female groups males often compete to attain and maintain a high dominant rank, and consequently to access to breeding females. Male testosterone levels and aggression rates are higher during periods of social instability, rather than during the mating season (Beehner et al. 2006; Cavigelli and Pereira 2000). Also, other authors found that in species with low aggression levels and little competition associated with access to mates (e.g., muriquis, *Brachyteles arachnoides*: Strier, Ziegler, and Wittwer 1999; moustached tamarins, *Saguinus mystax*: Huck et al. 2005), testosterone fluctuations are not associated with the breeding season. Although aggressive interactions have occasionally been observed in male gentle lemurs competing for a fertile female (Haring and Davis 1998), the social stability and lack of male competitors in our study groups may explain the absence of substantial fluctuations in testosterone levels. Captive data suggest that relatively low concentrations of circulating testosterone are generally adequate to maintain male reproductive function (Dixson 2012), avoiding the unnecessary costs of sustaining elevated testosterone levels (Wingfield et al. 1999). Obviously, another possible explanation is that our scent enrichment did not fully reflect the mixture of chemical compounds conveying information about female fertility to trigger testosterone in the male lemurs to rise.

## 5 Research Limitations

We acknowledge some major limitations that could have impacted on the efficacy of the scent enrichment we created. Our subject sample size was small (i.e., one successful breeding female, four unsuccessful breeding pairs). Additionally, it would have been crucial to verify whether the females we tested were not ovulating during the study period. Moreover, the synthesized odour mixture might not have faithfully signalled ovulation due to difficulties with odour sampling (i.e., lack of odour secretions on the anogenital area of the breeding female and swab contaminants potentially covering lemur compounds) and anogenital samples coming from one female during one breeding season. Finally, due to the small pool of odour samples, we had to mix the compounds we synthesized 1:1 in proportion, which does not reflect the real ratio of the chemical mixture released via anogenital odour by the fertile female lemurs.

## 6 Conclusions and Perspectives

In conclusion, our study was the first that aimed to stimulate naturalistic behaviors in captive Alaotran gentle lemurs using a resynthesized biologically meaningful scent as olfactory enrichment. Our findings show that natural biologically relevant odour have the potential to induce species‐specific behaviors, particularly sexual behavioral patterns. To enhance the efficacy of this scent enrichment, we must conduct further investigations of both the volatile and volatile components of the chemical profile of ovulatory female Alaotran gentle lemurs and enlarge the sample size when testing the refined scent mixture on male conspecifics.

## Author Contributions

**Sara Fontani:** study conception and design, training of research assistants in data collection and endocrinological laboratory work, writing original draft, funding acquisition, project management. **Gale Glendewar:** study conception and design, training of research assistants in data collection. **Rachel Cowen:** study conception and design, training of research assistants in data collection. **Georgia Callagan:** data collection and analysis. **Anna B. Costantini:** data collection and analysis. **Emily Elwell:** statistical analysis, writing resubmitted draft. **Colin Dubreuil:** statistical analysis, writing original and resubmitted drafts. **Matthew Palframan:** chemical laboratory work. **Stefano Vaglio:** study conception and design, chemical laboratory work, writing and reviewing original and resubmitted drafts, funding acquisition, project administration, supervision. All authors read and approved the final manuscript.

## Ethics Statement

The study followed the institutional and international guidelines for the care and use of captive animals, involving noninvasive methods for obtaining behavioral data and fecal samples from the gentle lemurs. Specifically, the study was conducted in compliance with the CITES and approved by the Life Sciences Ethics Committee at University of Wolverhampton (United Kingdom of Great Britain and Northern Ireland, UK) (REC number LSEC/202021/SV/52) and the Ethics Committees at Jersey Zoo (Channel Islands), Parc Zoologique & Botanique de Mulhouse (France), Birmingham Wildlife Conservation Park, and Zoological Society of London (ZSL) London Zoo (UK). Our research work was consistent with the ARRIVE guidelines for ethical treatment of Nonhuman primates and abided by the American Society of Primatologists Principles for the Ethical Treatment of Nonhuman Primates.

## Conflicts of Interest

The authors declare no conflicts of interests.

## Supporting information

Supporting information.

## Data Availability

The datasets are available on the Open Science Framework (OFS) repository with following persistent identifier – DOI: 10.17605/OSF. IO/PFT7C. We also confirm that the datasets have a CC0 Public Domain Dedication license applied.
