# Supplementary material for: Novel Scent Enrichment Enhances Socio‐Sexual and Olfactory Behaviors in Zoo‐Housed Gentle Lemurs
Source: Am J Primatol. 2024 Dec 18;87(1):e23716. doi: 10.1002/ajp.23716 (PMC11655772; doi:10.1002/ajp.23716)
Supplement: Supplementary file 1 — Supporting information. [file AJP-87-e23716-s001.docx]

| **Formula:** | | | | |
| --- | --- | --- | --- | --- |
| Olfactory behave count ~ Enrichment Phase + sex + zoo + offset(log(observation time)) + (1 \| Subject ID) | | | | |
| **AIC** | **BIC** | **logLik** | **deviance** | **df.resid** |
| 1344.5 | 1374.4 | -663.2 | 1326.5 | 198#7 |
| **Scaled Residuals:** | | | | |
| **Min** | **1Q** | **Median** | **3Q** | **Max** |
| -1.4003 | -0.6491 | -0.2678 | 0.5733 | 5.5028 |
| **Random Effects:** | | | | |
| Groups | Name | Variance | Std.Dev. |  |
| Subject ID | Intercept | 0.0812 | 0.2849 |  |
| Number of obs: 206, groups:  Subject ID, 8 | | | | |
| **Fixed Effects:** | | | | |
| Predictors | Estimate | Std. Error | z value | Pr(>\|z\|) |
| Intercept | -4.0577 | 0.2638 | -15.379 | <2e-16 |
| Enrichment: During | 0.3046 | 0.1354 | 2.249 | 0.024489 |
| Enrichment: Post | 0.2643 | 0.1175 | 2.249 | 0.024533 |
| Sex: Male | 0.8046 | 0.2263 | 3.555 | 0.000378 |
| Zoo: Jersey | 0.4104 | 0.3183 | 1.289 | 0.197324 |
| Zoo: London | 0.3781 | 0.3228 | 1.171 | 0.241482 |
| Zoo: Mulhouse | 0.2571 | 0.3194 | 0.805 | 0.420746 |
| Marginal R: 0.058, Conditional R: 0.081 | | | | |

**Table A1**. Olfactory full model output.

| **Formula:** | | | | |
| --- | --- | --- | --- | --- |
| Sex behave count ~ Enrichment Phase + sex + zoo + offset(log(observation time)) + (1 \| Subject ID) | | | | |
| **AIC** | **BIC** | **logLik** | **deviance** | **df.resid** |
| 562.2 | 592.2 | -272.1 | 544.2 | 197 |
| **Scaled Residuals:** | | | | |
| **Min** | **1Q** | **Median** | **3Q** | **Max** |
| -1.3321 | -0.7736 | -0.3487 | 0.4578 | 5.2238 |
| **Random Effects** | | | | |
| Groups | Name | Variance | Std.Dev. |  |
| Subject ID | Intercept | 0.4291 | 0.6551 |  |
| Number of obs: 206 groups:  Subject ID, 8 | |  |  |  |
| **Fixed Effects** | | | | |
| Predictors | Estimate | Std. Error | z value | Pr(>\|z\|) |
| Intercept | -5.59456 | 0.55217 | -10.132 | <2e-16 |
| Enrichment: During | 0.87779 | 0.18543 | 4.732 | 2.2e-06 |
| Enrichment: Post | 0.61250 | 0.17530 | 3.494 | 0.000476 |
| Sex: Male | -0.32813 | 0.49943 | -0.657 | 0.511170 |
| Zoo: Jersey | -0.58298 | 0.68614 | -0.850 | 0.395512 |
| Zoo: London | -0.07766 | 0.72300 | -0.107 | 0.914462 |
| Zoo: Mulhouse | -0.59564 | 0.68498 | -0.870 | 0.384536 |
| Marginal R: 0.029, Conditional R: 0.114 | | | | |

**Table A2**. Sexual full model output.

|  | | | | |
| --- | --- | --- | --- | --- |
| testosterone ~ category + zoo | | | | |
|  | | | | |
| **Min** | **1Q** |  | **Max** |  |
| -11.8765 | -3.5629 |  | 16.5295 |  |
| **Coefficients** | | | | |
| Predictors | Estimate | Std. Error | t value | Pr(>\|t\|) |
| Intercept | 34.593 | 1.431 | 24.175 | <2e-16 |
| Enrichment: During | -1.008 | 1.451 | -0.695 | 0.489 |
| Enrichment: Post | -1.192 | 1.326 | -0.899 | 0.371 |
| Zoo: Jersey | -16.546 | 1.613 | -10.260 | <2e-16 |
| Zoo: London | -8.252 | 1.769 | -4.664 | 1.11e-05 |
| Zoo: Mulhouse | -12.529 | 1.587 | -7.894 | 8.11e-12 |
| Multiple R squared: 0.5683, Adjusted R squared: 0.5435 | | | | |

**Table A3**. Testosterone full model output.

| **Formula:** | | | | | |
| --- | --- | --- | --- | --- | --- |
| log(cortisol) ~ enrichment phase + sex + zoo + (1 \| Subject ID) | | | | | |
| **Scaled Residuals:** |  |  |  |  |  |
| **Min** | **1Q** | **Median** | **3Q** | **Max** |  |
| -3.7049 | -0.6281 | -0.0135 | 0.5728 | 2.8061 |  |
| **Random Effects** | | | | | |
| Groups | Name | Variance | Std.Dev. |  |  |
| Subject ID | Intercept | 0.008701 | 0.09328 |  |  |
| Residual |  | 0.117754 | 0.34315 |  |  |
| Number of obs: 180, groups:  Subject ID: 8 | | | | | |
| **Fixed Effects** | | | | | |
| Predictors | Estimate | Std. Error | df | t value | Pr(>\|t\|) |
| Intercept | 4.479033 | 0.099455 | 4.082171 | 45.036 | 1.16e-06 |
| Enrichment: During | -0.007778 | 0.064088 | 170.468705 | -0.121 | 0.90354 |
| Enrichment: Post | -0.058331 | 0.060202 | 170.434739 | -0.969 | 0.33395 |
| Sex: male | -0.092685 | 0.083834 | 3.199348 | -1.106 | 0.34505 |
| Zoo: Jersey | -0.747997 | 0.117928 | 3.154015 | -6.343 | 0.00681 |
| Zoo: London | -0.104819 | 0.121918 | 3.604983 | -0.860 | 0.44333 |
| Zoo: Mulhouse | -0.643256 | 0.1163169 | 2.989835 | -5.530 | 0.01176 |
| Marginal R: 0.464, Conditional R: 0.501 | | | | | |

**Table A4**. Cortisol full model output.
